# Correlation between age, testosterone and adiponectin concentrations, and sperm abnormalities in Simmental bulls

**DOI:** 10.14202/vetworld.2021.2124-2130

**Published:** 2021-08-18

**Authors:** Abdullah Baharun, Syahruddin Said, Raden Iis Arifiantini, Ni Wayan K. Karja

**Affiliations:** 1 Biology Reproductive Program, Post Graduate School, IPB University, Jl. Raya Dramaga, Dramaga Campus, Bogor 16680, Indonesia; 2 Animals Science Program, Faculty of Agriculture, Djuanda University, Jl. Tol Jagorawi No.1, Ciawi, Bogor 16720, Indonesia; 3 Research Center for Biotechnology, Indonesia Institute of Sciences (LIPI), Jl. Raya Jakarta-Bogor, Cibinong, Bogor 16911, Indonesia; 4 Department of Clinic, Reproduction, and Pathology, Faculty of Veterinary Medicine, IPB University, Jl. Agatis, Dramaga Campus, Bogor 16680, Indonesia

**Keywords:** adiponectin, age, Simmental bull, sperm abnormalities, testosterone

## Abstract

**Background and Aim:**

Capacity for sperm production is affected by age, which is related to the morphology of sperm abnormalities and can affect fertility. The aim of this study was to evaluate the relationship between age and concentrations of testosterone and adiponectin with sperm abnormalities in Simmental bulls.

**Materials and Methods:**

The study used 11 bulls, separated into three groups. The first group consisted of five bulls aged 4-5 years, and the second and third groups each consisted of three bulls, aged 6-7 and 8-10 years, respectively. The average sperm motility of the animals ranged from 57.66±2.60% to 70.17±0.22%. Blood samples were obtained from the coccygeal region of the animals. Testosterone and adiponectin analysis was performed using the enzyme-linked immunosorbent assay method. Sperm morphology was evaluated using carbol fuchsin-eosin staining according to the Williams method. Finally, correlations between testosterone and adiponectin concentrations, age, and sperm abnormalities were analyzed using Pearson’s correlation analysis.

**Results:**

The findings revealed a significant correlation (p<0.01) between the concentrations of testosterone and adiponectin (−0.538), age (−0.588), and abnormal sperm morphology (−0.912). Moreover, they revealed that the concentration of testosterone in the bulls aged 8-10 years was lower, at 21.89±4.56 ng/mL, compared to that in the bulls aged 4-5 years, at 36.15±1.29 ng/mL, and 6-7 years, at 35.16±5.39 ng/mL. The findings also revealed a positive correlation between adiponectin concentration and age (0.529) and sperm abnormalities (0.506). The increase in testosterone concentration was inversely related to the adiponectin concentration (−0.538). Moreover, the mean amount of abnormal sperm increased with increasing age: 3.82±0.33% in the group aged 4-5 years, and 4.40±0.72% and 10.20±1.97% in the groups aged 6-7 years and 8-10 years, respectively.

**Conclusion:**

The study data indicate that there is a decrease in testosterone concentration, a high adiponectin concentration, and an increase in abnormal sperm with increasing age in bulls.

## Introduction

Application of bull semen through artificial insemination (AI) can increase productivity and the rapid distribution of bulls of outstanding genetic quality and with optimized function [1]. Moreover, the combination of AI with the strict selection of bulls in livestock breeding programs plays a strategic role in producing high-quality offspring with enhanced performance. Systems for selecting prospective bulls can be implemented using the breeding soundness examination (BSE) [2]. The BSE application used most often for selecting Simmental bulls entails measuring their scrotal circumference and health status. However, the parameters of semen analysis that is used remain limited, focusing only on examining the concentration and motility of sperm to determine the number of diluent levels. Another parameter that should be considered for examination is sperm morphology, which may increase sperm abnormalities [3].

Assessment of sperm morphology is essential in determining the sperm-oocyte fertilization capacity [4]. According to Felton-Taylor *et al*. [5], sperm abnormalities reduced the fertilization rate and the ability of sperm to penetrate the zona pellucida. These abnormalities increased in older bulls, possibly due to either low testosterone hormone concentration [6] or low adiponectin concentration [7]. Due to this condition, seminiferous germinal tubules decreased, which play an essential role in spermatogenesis [3]. Hafizuddin *et al*. [7] stated that testosterone concentration, including sperm abnormalities, was not correlated with age. However, either high or low concentrations of testosterone can affect the morphological formation (normal or abnormal) of sperm [8] through the hypothalamus-pituitary-gonad axis mechanism, which is important for spermatogenesis [9].

In steroidogenesis, testosterone production is linked to adiponectin [10]. Messenger ribonucleic acid (mRNA) for adiponectin has been found in the testicles and Leydig cells [11] and in spermatocytes [12] had multiple roles interact with the other hormone to induce the target genes. Adiponectin can stimulate the germinal cells of lactate by activating adenosine monophosphate-activated protein kinase (AMPK). AMPK maintains the integrity of the functional complex between Sertoli and germinal cells for maintaining the microenvironment for spermatogenesis [13]. Moreover, the capacity for sperm production is influenced by age, which is related to the morphology of sperm abnormalities and can affect fertility [14].

The above findings indicate the need for study of the relationship between testosterone and adiponectin concentrations and age with morphological abnormalities of sperm. This study evaluated the relationship between age, testosterone and adiponectin concentrations, and sperm abnormalities in Simmental bulls to investigate various sperm parameters. In addition, this study was used by AI center as a parameter of correct semen evaluation for the selection of prospective bulls in Indonesia.

## Materials and Methods

### Ethical approval

The Animal Ethics Commission of IPB University approved the animal models and experimental designs for this study with certificate number 158-2019 IPB.

### Study period and location

This study was conducted from October 2019 to July 2020. It used semen and blood obtained from Central Java Artificial Insemination (AIC) in Ungaran Central Java. Sperm morphology was evaluated at the Reproductive Rehabilitation Unit, Division of Reproduction and Obstetrics, Department of Veterinary Clinic, Reproduction and Pathology, Faculty of Veterinary Medicine, IPB University, Bogor, Indonesia. Concentrations of testosterone and adiponectin were measured using the enzyme-linked immunosorbent assay (ELISA), which was performed according to the manufacturer’s protocol at the Primate Research Center, IPB University.

### Animals

The semen samples were obtained from 11 Simmental bulls aged between 4 and 10 years, selected from AIC in Ungaran Central Java. The bulls were maintained according to the standard operating procedures of the AI Center. They were housed individually in pens equipped with a supply of food and water. Each bull was fed 10% fresh forage and 1% concentrate based on their body weight daily. They were fed twice a day, in the morning and evening, and water was provided *ad libitum*.

We divided the bulls into three age groups: 4-5 years (five bulls), 6-7 years (three bulls), and 8-10 years (three bulls), with the average motility of sperm from fresh semen ranging from 57.66±2.60% to 70.17±0.22%. Sperm motility based on the data of the Central Java AIC frozen semen production from 2018 to 2019 was used.

### Data collection and semen quality testing

Data from direct observation confirmed the secondary data of quality reports of fresh semen obtained from 2018 to 2019. The semen was collected in the morning twice a week using an artificial vagina. The semen quality was then evaluated both macroscopically and microscopically. The microscopic assessment was conducted by examining the mass motility and the morphology of the sperm using the technique from Baracaldo *et al*. [15]. Sperm motility was assessed using an Olympus CX23 microscope.

We obtained samples for the morphological testing of the sperm by making a smear of mixed semen and saline solution at a ratio of 1:4. The smear was air-dried. The process of staining sperm and observing sperm morphology was carried out at the Laboratory of the Reproductive Rehabilitation Unit, Faculty of Veterinary Medicine, IPB University. The samples were stained with carbol fuchsin-eosin based on the Williams method developed in 1920 and modified by Lagerlof in 1934 [16].

### Measurement of testosterone and adiponectin levels

Approximately 3-5 mL of blood was collected from the coccygeal region of the bulls, using a 5 mL disposable syringe containing ethylenediaminetetraacetic acid (Three Fingers, USA). The blood was then centrifuged at 3000 rpm for 10 min at room temperature (20°C). Subsequently, blood plasma was transferred immediately to a small tube and stored at −20°C for further analysis.

Testosterone was analyzed using the ELISA kit bovine testosterone (Signalway Antibody, #EK0019) and bovine adiponectin (Cusabio, Cat#SCB-E14054B). Blood plasma was diluted with distilled water at a ratio of 1:4. We then prepared standard solutions with concentrations ranging from 0.2 to 16 ng/mL. We transferred the sample and standard solutions (25 μL each) into the ELISA microplate well and added conjugate enzymes (except blank) before covering the microplate with cling film. The mixture was then homogenized for 10 s using a vortex and incubated for 60 min at room temperature (18°C). Each microplate well was washed 3-4 times with 300 μL of solvent, after which 200 μL of the substrate was added and incubated for 15 min. The reaction was stopped by adding 100 μL of stop solution to each microplate well. We then determined the absorbance at 450 nm using an ELISA reader [6].

### Statistical analysis

Data on the quality of the fresh semen from Simmental bulls were analyzed descriptively. The means of testosterone and adiponectin concentrations, age, and morphological abnormalities of sperm were analyzed using one-way analysis of variance, and p<0.05 was determined as being a significant difference. Correlations between the means of the groups’ testosterone and adiponectin concentrations with age and sperm abnormalities were determined using a Pearson’s correlation test. Finally, data were analyzed using Statistical Package for the Social Sciences version 20.0 software (IBM Corp., NY, USA).

## Results

### Age, sperm abnormalities, and testosterone and adiponectin concentrations

**Figure-1:**
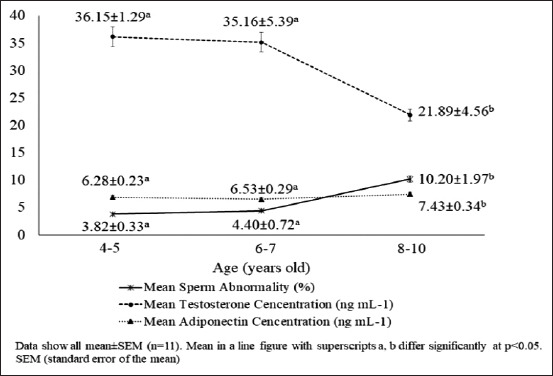
Bull age, sperm abnormalities, and testosterone and adiponectin concentrations in Simmental bulls.

The findings revealed that, on average, sperm abnormalities increased with age, with the increase being significant in the group aged 8-10 years (Figure-1). The average sperm abnormalities in accordance with the age groups were 3.82±0.33%, 4.40±0.72%, and 10.20±1.97%, respectively. The findings also revealed that Simmental bulls aged 4-7 years had a higher testosterone level (p<0.05) than did those aged 8-10 years (Figure-1). Meanwhile, there was a significant (p<0.05) increase in adiponectin concentration in the group aged 8-10 years compared to the other age groups (Figure-1).

**Figure-2:**
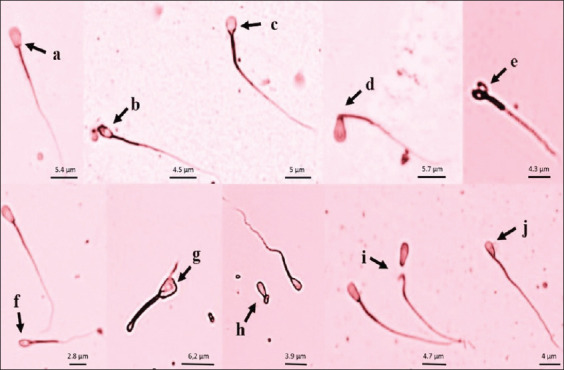
Morphology of sperm of Simmental bulls: (a) Normal sperm; (b) pear shaped; (c) round head; (d) detached head; (e) double head; (f) microcephalus; (g) macrocephalus; (h) dag defect; (i) undeveloped; (j) narrow at the base.

The findings indicated that there were differences (p<0.05) in the total variation of sperm abnormalities in the bulls (Table-1). Moreover, the sperm abnormalities in the bulls were found to be mainly primary (Figure-2).

**Table-1:** Types of sperm abnormalities in individual Simmental bulls.

Type of sperm abnormality	Bull identity										

	61,672	61,673	61,671	61,568	61,567	61,464	61,362	61,361	61,257	61,249	61,042
Pear shaped	0.80	-	0.80	1.40	0.40	0.40	0.80	0.80	2.60	4.60	1.20
Knobbed acrosome	-	-	-	-	-	-	-	-	-	-	-
Narrow at the base	-	-	-	-	-	-	-	0.20	-	-	-
Narrow (tapered head)	-	-	-	-	-	-	-	0.20	-	-	-
Abnormal contour	-	-	-	-	-	-	-	-	-	-
Underdeveloped	-	0.60	0.80	0.80	0.40	0.80	-	0.80	-	1.00	0.80
Round head	-	-	0.20	0.30	-	-	-	1.00	-	-	0.40
Macrocephalus	0.20	-	-	-	-	-	0.20	-	0.80	0.40
Microcephalus	-	0.40	0.60	-	-	0.80	1.00	0.20	0.60	1.80	0.40
Double head	-	-	-	-	-	-	-	-	0.60	-	-
Abaxial	0.20	-	-	-	-	-	-	0.20	0.60	-	-
Knobbed acrosome defect	-	-	-	-	-	-	-	-	-	-	-
Detached head	-	0.60	0.20	0.80	0.60	0.20	0.40	0.80	-	0.80	1.00
Diadem	-	-	-	-	-	-	-	-	-	-	-
Proximal droplet	-	-	-	-	-	0.20	-	0.20	0.80	-	0.40
Pseudodroplet	0.20	-	-	-	-	-	-	-	-	-	-
Tail stump defect	-	-	-	-	-	-	-	0.40	-	-	-
Abnormal mid-piece	-	-	-	-	-	-	-	-	-	-
Fracture tail	-	-	-	0.20	-	-	0.40	0.60	-	0.20	0.20
Double tail	-	-	-	-	-	-	-	-	-	-	-
Bent tail	-	-	-	0.20	-	-	-	-	-	-	-
Coiled	2.40	2.60	1.40	0.80	1.20	1.00	1.40	-	4.60	4.60	2.20
Total sperm abnormalities	3.80±0.43^a^	4.20±0.52^a^	4.08±0.19^a^	4.50±0.16^a^	2.60 ±0.19^a^	3.40±0.14^a^	4.00±0.19^a^	5.80±0.09^a^	9.80±0.67^b^	13.80±0.70^b^	7.00±0.21^a^

Different letters following the numbers indicate significant differences (p<0.05)

### Correlation between age, sperm abnormalities, and testosterone and adiponectin concentrations

The findings revealed a positive correlation between adiponectin concentration and sperm abnormalities in the older Simmental bulls and a significant negative correlation between testosterone concentration and sperm abnormalities (Table-2). Finally, the difference in adiponectin concentration was significantly higher (p<0.05) in the group aged 8-10 years compared to the other age groups (Figure-1).

**Table-2:** Correlations between age, sperm abnormalities, testosterone concentration, and adiponectin concentration.

Variable	Age	Sperm abnormalities	Testosterone concentration	Adiponectin concentration
Age	1	0.637*	−0.588*	0.529*
Sperm abnormalities	0.637*	1	−0.912*	0.506*
Testosterone concentration	−0.588*	−0.912*	1	−0.538*
Adiponectin concentration	0.529*	0.506*	−0.538*	1

* A significant correlation (p<0.01)

## Discussion

As bulls grow, their testosterone concentrations increase, and so do the chances of sperm abnormalities. The sperm abnormality assessed by the criteria used in this study was considered usual, in line with the findings of Parthipan *et al*. [17] for *Bos taurus*. However, the results of morphological analysis of sperm abnormalities and testosterone concentrations showed different outcomes (Figure-1). According to the Indonesian National Standard for frozen bull semen (SNI number: 4869.1: 2017) [18] and Agriculture Ministry Republic of Indonesia Regulation (10/Permentan/PK.210/3/2016) [19], the percentage of sperm abnormalities in fresh semen to be processed into frozen semen is <20%. In this study, all the semen were suitable for processing into frozen semen.

In each bull, the testosterone concentration was influenced by the physiology of the endocrine gland, which plays a key role in maintaining testicular functions for spermatogenesis. This indicates that the testes functioned normally and displayed a particular pattern. As shown in Figure-1, there was a correlation between decreased testosterone concentrations and an increased level of abnormal sperm in the group aged 8-10 years (10.20%). Our findings are in line with those of Dasrul *et al*. [6], who reported a relationship between testosterone concentration and semen quality (sperm motility and sperm abnormalities) in Aceh bulls and also crossbred cattle of Holstein Friesian×Tharparkar [20]. Several studies also have shown that low semen quality is associated with a deficiency in testosterone concentrations [21].

Our findings indicated that there were differences in the total variation of sperm abnormalities in the bulls (Table-1). Moreover, the sperm abnormalities in the bulls were found to be primary, as follows: Pear-shaped, narrow at the base, narrow (tapered head), double head, undeveloped, round head, variable size (macrocephalus/microcephalus), abaxial, and detached head (Figure-2).

The level of primary sperm abnormality was higher in the group of older bulls (8-10 years) (Table-1). Moreover, this increase was influenced by either dysfunction or decreased performance of the seminiferous tubules [3], which plays a significant role in spermatogenesis. Primary sperm abnormalities in the head and acrosome generally occur during spermatogenesis. Furthermore, follicle-stimulating hormone (FSH) and luteinizing hormone (LH) influence spermatogenesis and sperm maturation. LH modulates testosterone synthesis in Leydig cells and aromatizes estradiol in Sertoli cells. Finally, both high and low concentrations of testosterone can affect the morphological formation (normal or abnormal) of sperm [8].

As the ratio of primary to secondary abnormalities is 1:1, the number of primary abnormalities is not expected to exceed 10% [22] because a greater increase in percentage could affect fertility [23]. According to Nagy *et al*. [24], sperm abnormalities are considered a threat when they reach 18%-20% because such levels could reduce fertility. This is supported by Attia *et al*. [4], who showed that a bull cannot have high fertility when its percentage of abnormal sperm is >17 [4]. Low fertility rates due to high sperm abnormalities can hinder fertilization and the success of an AI program, contributing to bulls’ maintenance [4,25].

The findings demonstrated that age and testosterone concentrations were associated with an increase in sperm abnormalities. Compared to other bulls, the bulls with identity numbers 61,257 and 61,249 showed a higher level of sperm abnormalities (p<0.05), at 9.80% and 13.80%, respectively (Table-1). Moreover, the high levels of sperm abnormalities in the two bulls were associated with their increasing age (8-10 years) and lower concentrations of testosterone (23.27 ng/mL and 13.39 ng/mL) in blood plasma (Figure-1). According to Felton-Taylor *et al*. [5], the percentage of morphological abnormalities of *Bos indicus* and *B. taurus* sperm was correlated with age, season, and environment.

There was a correlation between the increase of sperm abnormalities and a decline in testosterone concentration (−0.912) (Table-2). Moreover, we found that older bulls (8-10 years) could have a reduced concentration of testosterone and an increased level of abnormal sperm. However, according to Attia *et al*. [4], the level of sperm abnormality in bulls in this age group was still expected to be 10.20%, on average,

The adiponectin concentration in bulls in the older group was significantly higher than were those in the two younger groups. As stated by Heinz *et al*. [25], the older the bulls, the higher the adiponectin concentration. In other words, as an animal grows older, adipose tissue is more prone to hypertrophy which leads to an increase in both adiponectin concentrations in the blood and insulin sensitivity within the tissue [26]. Increases and decreases in concentrations of adiponectin depend on adipose tissue insulin resistance factors, intra-abdominal fat, and the lipoprotein profile [27]. An increase of lipoproteins has been positively correlated with adiponectin concentrations [10].

We have also shown that an increase in adiponectin concentration can suppress testosterone production (−0.538). Adiponectin is a protein formed from 224 amino acids produced by adipose tissue [28] and is related to cases of insulin resistance and obesity. The process of the conversion of aromatic testosterone to estradiol in peripheral fatty tissue [29] and testes could reduce testosterone concentrations [30]. Meanwhile, low testosterone production over a long period leads to obesity that may later reduce sperm production capacity, as indicated by a positive relationship (p<0.01) between adiponectin concentration and increased morphological abnormalities in Simmental bovine sperm (0.506).

Our analysis (Table-2) implies a significant negative correlation (p<0.01) between sperm abnormality and testosterone concentration (−0.912). This suggests that high testosterone concentration contributes to the morphology of normally formed sperm. Testosterone maintained normal sperm morphology through the AMP-AMPK, increasing glucose uptake and glucose transporter 1 (GLUT1). GLUT1 plays a role in converting glucose to lactate [31]. The results of the conversion are transferred from Sertoli cells to germ cells through the protein-linked transporters MCT4 and MCT2 [13].

Maintaining the viability of the spermatocytes process caused lactate production in Sertoli cells. This prevents apoptosis during normal spermatogenesis to maintain the morphology of sperm [13]. Moreover, it may be assumed to contribute to increased testosterone production [32]. As confirmed by Smith and Walker [33], testosterone plays a role in the meiosis stage of germ cells during sperm formation.

Testosterone is involved in spermatogenesis, particularly the phase of sperm maturation [8]. This hormone works synergistically with FSH and prolactin. Interaction between LH and its receptors activates the adenyl cyclase system, protein kinase, and RNA synthesis [34]. Consequently, it increases the synthesis of pregnenolone, with 21 carbon chains (C21), from cholesterol (C27) by the mitochondria of Leydig cells. The final product of this process is the formation of testosterone (C19) [34], which results in spermatogenesis and is correlated with semen quality [20].

This study contradicts the conclusion of Hafizuddin *et al*. [7] that there is not a strong correlation between testosterone concentration and normal sperm morphology. Genetic factors also contribute to normal sperm morphology [35]. This has been proven by the existence of a genetic expression disorder that evokes sperm abnormalities [36]. Some types of sperm abnormalities are related to genetic expression [22]. However, the type of primary sperm abnormalities with genetic expression occurs only during spermatogenesis. Sperm abnormalities can be categorized based on either the location of the damage (head, mid-piece, and tail) or the origin (primary abnormality, in the testis; secondary abnormality, in the epididymis; and tertiary abnormality, in the gland accessories/post-ejaculation).

Our findings could be used as the basis for selecting bulls for the purpose of providing frozen semen, especially for bulls that are older than 10 years. Such evaluation is essential to prevent the spread of inferior quality sperm and a consequent decline in the quality of livestock. Moreover, older bulls may have increased sperm abnormalities leading to the presence of a defective gene [22].

## Conclusion

The study showed that from the age of 7 years, Simmental bulls started experiencing an increase in adiponectin and sperm abnormalities and a decline in testosterone concentration. Furthermore, morphological assessment of sperm and testosterone hormone analysis should be included in semen evaluation parameters for selecting a prospective bull.

## Authors’ Contributions

AB, RIA, and NWKK: Conceptualized and designed the experiment, conducted the literature review, and wrote the first draft of the manuscript. AB, SS, RIA, and NWKK: Edited and revised the draft of the manuscript. All authors read and approved the final manuscript.
